# Shelf Life and Sensory Profile of Potentially Probiotic Mead Obtained by Co-Fermentation of Water Kefir and *Saccharomyces boulardii*

**DOI:** 10.3390/foods15010099

**Published:** 2025-12-29

**Authors:** Handray Fernandes de Souza, Ricardo Donizete Teixeira, Fabiano Vaquero Silva Junior, Felipe Donizete Teixeira, Karina Nascimento Pereira, Adriano Gomes da Cruz, Igor Viana Brandi, Eliana Setsuko Kamimura

**Affiliations:** 1 Department of Food Engineering, School of Animal Science and Food Engineering, Universidade de São Paulo, Av. Duque de Caxias Norte, 225, Pirassununga 13635-900, SP, Brazil; ricardo.donizete@usp.br (R.D.T.); fabianovaquero@usp.br (F.V.S.J.); felipeteixeira1@usp.br (F.D.T.); karinascimento@usp.br (K.N.P.); elianask@usp.br (E.S.K.); 2 Department of Food Science and Technology, University of California, One Shields Avenue, Davis, CA 95616, USA; 3 Department of Food, Federal Institute of Science and Technology of Rio de Janeiro (IFRJ), Rio de Janeiro 20270-021, RJ, Brazil; adriano.cruz@ifrj.edu.br; 4 Institute of Agricultural Sciences, Federal University of Minas Gerais, Av. Universitária, 1000, Montes Claros 39404-547, MG, Brazil; ibrandi@ica.ufmg.br

**Keywords:** fermented alcoholic beverage, probiotic yeast, storage, sensory characteristics, acceptability

## Abstract

Mead is an alcoholic beverage obtained through the action of yeast in a diluted solution of honey and water. According to the literature, recent studies have demonstrated the potential of the probiotic yeast *Saccharomyces cerevisiae* var. *boulardii* in mixed fermentation with microbial sources such as kombucha and water kefir for the development of mead. Although mead produced by mixed fermentation of *S. boulardii* and water kefir has already been proposed, characteristics related to the shelf life and sensory profile of this product have not yet been evaluated. The present study aimed to determine the shelf life and sensory characteristics of potentially probiotic mead produced by mixed fermentation of *S. boulardii* and water kefir. The main results showed that mead produced by *S. boulardii* and water kefir has a shelf life of 60 days when stored at 7 ± 1 °C, with viable cell counts above 6 log CFU/mL for yeasts and lactic acid bacteria, meeting the minimum count recommended for probiotic foods. With regard to sensory characteristics, the product showed high sensory acceptability with scores above 7.0 points for all attributes evaluated and obtained a high purchase intention, with an average score of 5.17 points. In addition, mead by *S. boulardii* and water kefir was characterized by sensory attributes and terms described by panelists as effervescent, tasty, sweeter, refreshing, honey flavor, and less alcoholic. In conclusion, the potentially probiotic mead by mixed fermentation of *S. boulardii* and water kefir has an adequate shelf life and high sensory acceptance.

## 1. Introduction

Mead is an alcoholic beverage obtained through the action of yeast in a solution of honey and water [1,2,3,4,5,6,7,8]. Regarding its composition, the literature describes that meads are composed of various nutrients, including carbohydrates such as fructose, glucose, and others, minerals, vitamins, proteins, organic acids, phenolic compounds, and others [1,2,6,7,9,10].

According to Webster et al. [7], the last few decades have been marked by a resurgence of mead. Due to the growing number of artisanal producers, the drink has been gaining popularity and recognition, with mead even being included in the product lines of major beverage brands. In this sense, the commercial introduction of mead has favored new research focuses directed at areas that affect the quality of the mead produced, such as physical-chemical characteristics, aromatic profiles, and sensory characterization, in addition to potential health benefits, for example, improved profiles of bioactive compounds such as phenolics and antioxidants, and the inclusion of probiotic yeast strains [3,5,6,8,9,10,11,12].

In the context of incorporating probiotic yeasts into foods and beverages, *Saccharomyces cerevisiae* var. *boulardii* (*S. boulardii*) stands out as the most widely investigated strain [10,13]. Among its probiotic properties, its therapeutic and preventive effects on gastrointestinal disorders are noteworthy, including the ability to reduce episodes of diarrhea, inhibit the growth of pathogenic bacteria, prevent the adhesion of microorganisms and toxins to the intestinal mucosa, and modulate the microbiota through competitive exclusion mechanisms, stimulates the production of immunoglobulins and other immune mediators, contributing to the regulation of local and systemic immune responses, reinforcing its potential as a functional microorganism [5,6,10,14,15]. In this sense, recent studies have shown that *S. boulardii* yeast, as a single starter or in mixed fermentation with kombucha and water kefir, has emerged as a potential alternative in the production of mead, providing innovative, differentiated products and a source of probiotics [5,6,10,12]. Along the same lines of research, recent efforts have shown the potential for applying immobilized and freeze-dried kefir cultures on natural supports, without cryoprotectants, for the simultaneous alcoholic and malolactic fermentation of cider at various temperatures (5–45 °C), enabling the development and/or production of low-alcohol or “mild” cider [16]. In another study, for example, immobilized kefir culture was evaluated and compared to free cells, which showed potential for the production of semi-dry and sweet wines at high temperatures [17]. However, knowledge about the shelf life and sensory profile of mead with *S. boulardii* and/or mixed fermentation with other microbial sources such as kefir is still scarce, and studies should be encouraged in this area of research.

With regard to the production of mead by *S. boulardii*, a recent study proposed the development of mead by mixed fermentation of probiotic yeast *S. boulardii* and water kefir [6]. In this study, the authors demonstrated that the combination of 10 g/L of water kefir grains and 0.75 g/L of *S. boulardii*, fermented for 9 days at a temperature of 25 °C, produced a probiotic mead with viable cells exceeding 8 log CFU/mL of *S. boulardii* and also for lactic acid bacteria (LAB), respectively. It should also be added that the product is a source of total phenolic compounds and antioxidants. However, knowledge about the shelf life and sensory characteristics of the mead developed by Souza et al. [6] has not yet been clarified, and studies in this line should be carried out in order to provide clearer and more valuable information that may contribute to its future introduction and commercialization in the market.

Therefore, this study aimed to assess the shelf life and sensory attributes of a potentially probiotic mead produced via mixed fermentation using *S. boulardii* and water kefir. The findings are expected to provide relevant technological and sensory insights to support the potential future introduction of this beverage into the fermented alcoholic products market.

## 2. Materials and Methods

For the development of this study, specifically the study of shelf life and sensory analysis, new batches of mead were produced based on the mixed fermentation of *S. boulardii* and commercial *S. cerevisiae*, with kefir from a sugar solution with brown sugar, as described by Souza et al. [6]. Thus, the methodologies used were described in detail.

### 2.1. Obtaining and Maintaining Microorganisms

*Saccharomyces cerevisiae* var. *boulardii* CCT 4308 (UFPEDA 1176) was obtained from the microorganism culture bank of the André Tosello Foundation (FAT, research and technology, Brazil) and cultivated as described by Souza et al. [6]. Water kefir was obtained from the culture bank of the Bioprocess Engineering Laboratory (FZEA/USP) and maintained in brown sugar solution (5%, *w*/*v*) at a temperature of 35 °C, with the brown sugar solution being replaced every 48 h, according to Souza et al. [6]. Under the described cultivation conditions, water kefir has the following physical-chemical and microbiological characteristics: soluble solids of 4 °Brix, pH 3.22, total acidity of 1.07% (% lactic acid), and lactic acid bacteria (LAB) count of 6.33 Log CFU/mL [6].

### 2.2. Mead Production

Mead production was carried out according to Souza et al. [6]. *S. boulardii* CCT 4308 (UFPEDA 1176) and kefir grains from a sugar solution with brown sugar (5%, *w*/*v*) were purchased and cultivated as described by Souza et al. [6]. In general, the amounts of honey and water were calculated, and the initial must was standardized to 25 °Brix of soluble solids [5,6]. This first mixture was pasteurized at 65 °C for 30 min and then cooled to 25 °C. Immediately afterwards, 0.75 g/L of *S. boulardii* and 10 g/L of water kefir grains were added to the must. Considering the commercial yeast *Saccharomyces cerevisiae* Mangrove Jack’s M05, a standard mead was also prepared using the same amounts of microorganisms, namely 0.75 g/L of *S. cerevisiae* and 10 g/L of water kefir grains. Three liters of mead were produced in polypropylene buckets (5 L volume), which were closed with lids containing an airlock device to maintain anaerobic conditions inside. The fermentation buckets were incubated in a BOD incubator (model 347 CD, São Paulo, Brazil, MERSE) and fermentation occurred at 25 °C for a period of 9 days. Three replicates were performed for each treatment, totaling six fermentation buckets. At the end of fermentation, the water kefir grains were removed from the fermentation buckets by filtration, and the mead was bottled in transparent glass bottles (previously cleaned and sanitized) with a volume of 275 mL. The bottles were then stored under refrigeration at a controlled temperature of 7 °C ± 1 °C. A total of 54 bottles were stored, 27 of which contained mead made with *S. boulardii* and kefir, and the other 27 bottles contained mead made with *S. cerevisiae* and kefir.

### 2.3. Shelf Life Analysis

To study the shelf life of the meads, physical-chemical and microbiological analyses were performed every 10 days, including analyses for pH, total acidity, soluble solids, alcohol content, viable yeast and lactic acid bacteria cell counts, and contaminating microorganisms for total coliforms, *Escherichia coli*, and *Salmonella* spp. Phenolic compounds and antioxidants were analyzed on the first and last days of storage. For greater clarity, new bottles were used for sampling every 10 days, with a total of six bottles being opened, three of which contained mead with *S. boulardii* and kefir and the other three with *S. cerevisiae* and kefir. The analyses were performed in triplicate for each bottle opened.

#### 2.3.1. Physical-Chemical Analyses

pH analyses were performed using a benchtop pH meter (model PG 1800, FARMA, São Paulo, Brazil), which was previously calibrated with pH 4 and 7 buffers. Total acidity was determined by titration with sodium hydroxide (0.1 N), using 10 mL of samples and phenolphthalein (1%) as an indicator. Soluble solids (°Brix) were analyzed using a portable refractometer (model RSG-100ATC, Rainsun, Shanghai, China), in which distilled water was used to calibrate the device for soluble solids equal to zero. The alcohol content (%) was analyzed using an ebulliometer (Kit-0700, CIENLAB, Campinas, Brazil), as described by Souza et al. [6]. All analyses were performed in triplicate.

#### 2.3.2. Phenolic Compounds and Antioxidants

For the analysis of total phenolic compounds, the method described by Everette et al. [18] and adopted by Souza et al. [6] was used. Initially, a standard curve of gallic acid was prepared with concentrations ranging from 0.01–0.05 mg/mL. Next, the mead samples were prepared, and the absorbance was read at 700 nm in a spectrophotometer (model 7305, Bibby Scientific Ltd., Stone, UK). Finally, the results obtained were expressed in mg of gallic acid equivalent per 100 mL of mead. All analyses were performed in triplicate.

Antioxidant potential was determined by ferric reducing antioxidant power (FRAP) [19] and 2,2′-azinobis-(3-ethylbenzothiazoline-6-sulfonic acid) (ABTS) [20], as adopted by Souza et al. [6]. For FRAP, a standard curve of trolox equivalents with concentrations ranging from 2.5 to 15 μmol/L was prepared in advance. The samples were prepared and read at an absorbance of 593 nm in a spectrophotometer (model 7305, Bibby Scientific Ltd., Stone, UK). Finally, the results obtained were expressed as μmol trolox equivalent per 100 mL. For ABTS, a standard curve of trolox equivalents with concentrations ranging from 100–2000 μmol/L was prepared in advance. Next, a 30 μL volume of the mead sample was added to 3.0 mL of the ABTS radical solution in a low-light environment, and the absorbance of the samples was read at 734 nm in a spectrophotometer (model 7305, Bibby Scientific Ltd., Stone, UK) after a 6 min reaction time. Finally, the results obtained were expressed as μmol trolox equivalent per 100 mL. All analyses were performed in triplicate.

#### 2.3.3. Viable Yeast Cell Count

The methodology described by Souza et al. [6] was used to count viable yeast cells, with modifications. Mead samples were subjected to serial dilutions, using 1 mL of mead to 9 mL of sterile peptone water (0.1%). Immediately afterwards, approximately 100 µL of diluted sample was spread over the surface of plates containing rose bengal agar base medium (KASVI, K25-610237, Merck Life Science S.L.U., Madrid, Spain). The plates were then incubated at 35 °C for 48 h. The viable yeast count was performed directly on the plates, and the results were expressed as Log CFU/mL. All analyses were performed in triplicate.

#### 2.3.4. Counting Viable Lactic Acid Bacteria (LAB) Cells

The methodology described by Souza et al. [6] was used to count viable lactic acid bacteria cells. Initially, approximately 1 mL of mead samples were subjected to serial dilutions in 9 mL of sterile peptone water (0.1%). Next, 1 mL of diluted sample was inoculated deeply, followed by a double layer of De Man, Rogosa, and Sharpe (MRS) (KASVI, K25-1043, Liofilchem, Roseto degli Abruzzi, Spain) medium in sterile Petri dishes. Immediately afterwards, the dishes were incubated upside down at 37 °C for 48 h. The lactic acid bacteria count was performed directly on plates and the results expressed in Log CFU/mL. All analyses were performed in triplicate.

#### 2.3.5. Total Coliform, *Escherichia coli*, and *Salmonella* spp. Analyses

Total coliform and *Escherichia coli* analyses were performed using compactDry™ EC microbiological kits (R-Biopharm AG, Compact Dry EC, Tokyo, Japan). Approximately 1 mL of pure sample, in addition to 10^−2^ and 10^−3^ dilutions, were deposited in the center of the Compact Dry plate surface and dispersed automatically and homogeneously. The plates were then incubated at 37 ± 1 °C for 24 ± 2 h. To interpret the results, it should be noted that *E. coli* forms blue/purple colonies, while coliforms acquire a red/pink color on the plates. The total coliform count is considered to be the sum of the red/pink and purple/blue colonies. CompactDry™ SL microbiological kits (R-Biopharm AG, Compact Dry SL, Tokyo, Japan) were used for the analysis of *Salmonella* spp. Thus, initially, 25 mL of the samples were pre-enriched in Lactated Ringer’s Broth (LRB) (KASVI, K25-1206, made in Spain) and incubated at 35 ± 2 °C for 24 ± 2 h. Then, 1 mL of sample was deposited in the center of the Compact Dry plate surface and dispersed automatically and homogeneously. *Salmonella* spp. colonies were counted based on color characteristics, such as individual or mixed black/green and/or normal blue/purple to yellow colonies. All analyses were performed in triplicate.

### 2.4. Sensory Analysis

For the sensory analysis, approximately 120 tasters were invited on a voluntary basis, consisting of untrained men and women of legal drinking age, which according to Brazilian law is set at 18 years of age. The sensory analysis was conducted at the University of São Paulo, Fernando Costa Campus, in the city of Pirassununga, São Paulo, and the tasters were students, employees, and/or visitors. Before conducting the sensory tests, all volunteers were informed about the objectives of the study and clarified regarding the ethical procedures adopted. The volunteers who agreed to participate in the study signed an informed consent form, giving them the right to withdraw from the study at any time without penalty. Before conducting the sensory analysis, microbiological analyses of the product were performed, confirming the absence of *Escherichia coli* and *Salmonella* spp., and negative results for coliforms at 35 °C and 45 °C. To standardize environmental conditions, the sensory analysis was conducted in a sensory analysis laboratory, using individual booths with white light and a controlled ambient temperature of 28 ± 1 °C.

During the sensory analysis, 20 mL of mead was served, with each sample being presented one at a time to each taster. The mead was kept refrigerated at a temperature of 7 °C ± 1 °C until serving. Random three-digit numbers were used to identify the samples. In addition, mineral water and unsalted crackers were provided to the tasters to eliminate possible interference between samples. The sensory analysis consisted of three samples of mead, which were designated as T1 (T1 = mead produced by mixed fermentation of *S. boulardii* and water kefir), T2 (T2 = mead from mixed fermentation of *S. cerevisiae* and water kefir), and T3 (T3 = commercial mead from the domestic market, a beverage made from eucalyptus flower honey). To perform the sensory analysis, the study was previously approved by the Human Research Ethics Committee of FZEA/USP, with a favorable opinion No. 12 and registered on the Brazil Platform under number CAAE 68971123.9.0000.5422, with the date of approval being 11 July 2023.

#### 2.4.1. Acceptance and Purchase Intention Test

The acceptance and purchase intention tests were conducted according to Souza et al. [12]. For the acceptance test, a structured 9-point verbal hedonic scale was used, ranging from “extremely dislike” (number 1) to “extremely like” (number 9), with an intermediate point of “don’t like nor dislike” (number 5). The attributes evaluated were overall impression, color, aroma, flavor, and alcohol content of the product. In the purchase intention test, a 7-point verbal attitude scale was used, with terms ranging from “would never buy” (number 1) to “would always buy” (number 7), with an intermediate point for the term “might buy/might not buy” (number 4).

#### 2.4.2. Check-All-That-Apply (CATA) Test

For the CATA test, words and terms associated with the sensory characteristics of mead were selected from studies in the scientific literature [6,12,21,22]. The selected terms are described below: yellowish color, brownish color, clear, cloudy, honey flavor, more alcoholic, less alcoholic, refreshing, sweeter, less sweet, dry, acidic, vinegary, tasty, effervescent, carbonated, fermented Flavor, shiny, bitter, sparkling. The tasters were then asked to indicate the terms that adequately characterized their experience and perception of the sample evaluated.

### 2.5. Statistical Analysis

A completely randomized design (CRD) was adopted for the study of the shelf life of mead. The parameters analyzed were pH, total acidity, soluble solids, alcohol content, viable yeast and lactic acid bacteria cell count, phenolic compounds, and antioxidants. All analyses were performed in triplicate. The results obtained were tabulated and expressed as mean ± standard deviation. Next, analysis of variance (ANOVA) was performed, followed by Tukey’s test with a 95% confidence level (*p* < 0.05), using RStudio software, version [64-bit] R-4.3.1 (Copyright © 2001–2023 R Core Team).

For the hedonic test of acceptance (overall impression, color, aroma, flavor, and alcohol content) and purchase intention, a one-way analysis of variance (ANOVA) was performed to verify whether there were significant differences between treatments for each sensory attribute. When a significant effect was detected, Tukey’s test with a 95% confidence level (*p* < 0.05) was applied. The hedonic test analyses were performed using Microsoft Excel^®^ 365.

For the CATA (Check-All-That-Apply) test, the chi-square test of independence was applied to identify statistically significant associations between sensory attributes and the different samples evaluated. Statistical analysis was performed using Microsoft Excel^®^ 365, adopting a confidence level of 95% (*p* < 0.05).

## 3. Results and Discussion

### 3.1. Shelf Life Results

**Figure 1:**
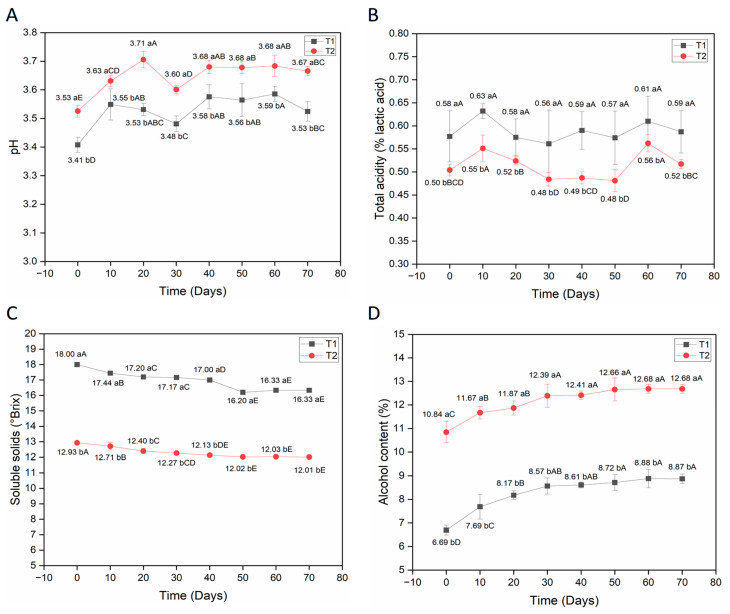
Physical-chemical parameters of mead during storage at 7 °C ± 1 °C. (**A**) pH, (**B**) Total acidity (% lactic acid), (**C**) Soluble solids (°Brix), and (**D**) Alcohol content (%). T1 = mead with kefir and *S. boulardii*; T2 = mead with kefir and commercial *S. cerevisiae*. Different lowercase letters indicate significant differences between treatments on the same day. Different uppercase letters indicate significant differences for each specific treatment and throughout the storage period (according to Tukey’s test, *p* < 0.05). All analyses were performed in triplicate.

Figure 1 shows the results of the pH, total acidity, soluble solids, and alcohol content parameters of mead during storage at 7 °C ± 1 °C for 70 days. According to Figure 1A, mead produced with probiotic yeast *S. boulardii* and water kefir (T1) and commercial yeast *S. cerevisiae* and water kefir (T2) show similar behavior for all parameters analyzed during storage. It is noted that the pH of T1 was statistically lower (*p* < 0.05) when compared to T2 during refrigerated storage (Figure 1A). On the other hand, the acidity results (Figure 1B) show that T1 had higher acidity during storage, with a statistical difference (*p* < 0.05) compared to T2. The pH and acidity results suggest greater synergy between *S. boulardii* and water kefir during storage, such that the microorganisms present are associated with greater conversion of fermentable sugars into organic acids, ensuring greater acidification of the product. In addition, mead made with commercial *S. cerevisiae* and kefir showed lower acidification during storage. It should be noted that the acidification of the developed meads is also related to water kefir in both treatments, due to the presence of different types of microorganisms, including yeasts and acetic and lactic bacteria, acting on fermentable sugars and contributing to the formation of acids, such as lactic and acetic acid [6,23,24].

With regard to soluble solids (Figure 1C), there was a significant reduction (*p* < 0.05) of 1.67 °Brix in mead with probiotic yeast *S. boulardii* and water kefir (T1), and 0.92 °Brix in mead with commercial yeast *S. cerevisiae* and water kefir (T2), when comparing the initial time (0 days) and 70 days of refrigerated storage. In contrast, a small increase in the alcohol content of the meads was observed during storage (Figure 1D). The alcohol content increased by 2.18% in T1 and 1.84% in T2, with a significant difference (*p* < 0.05) between the initial time (0 days) and 70 days of storage. The reduction in soluble solids (Figure 1C) and increase in alcohol content (Figure 1D) during storage show the existence of fermentative metabolic activity by the microorganisms *S. boulardii* and commercial *S. cerevisiae* in association with water kefir, which, although reduced, is still predominant under refrigerated storage conditions. This effect is not intentional and was expected, given that the product has high concentrations of viable cells, which are necessary for probiotic products containing live microorganisms. Thus, a slight continuous production of ethanol and a subtle decline in soluble solids (°Brix) were noted during refrigerated storage, which implies continuous metabolic activity in the bottled product, showing that microorganisms do not completely stop metabolic activity, but rather suddenly reduce it. According to Terhaag et al. [25], refrigerated storage does not prevent ethanol synthesis in beverages. These authors developed a probiotic lychee beverage with *Saccharomyces boulardii* and observed an increase in ethanol content in LB12 (lychee beverage supplemented with sucrose at 12 °Brix) during storage at 4 °C, possibly related to the continuous metabolism of yeast, even under refrigerated conditions.

It should also be noted that the values for soluble solids and alcohol content found in this study were slightly different from those observed by Souza et al. [6], although the values are generally similar. For example, the results for soluble solids found by Souza et al. [6] were 17.28 and 16.40 °Brix for mead with *S. boulardii* and water kefir (T1) and commercial *S. cerevisiae* and water kefir (T2), respectively, while values of 18.00 and 12.93 °Brix were observed in this study, respectively. For alcohol content, Souza et al. [6] observed values of 7.05 and 8.22% for mead with *S. boulardii* and water kefir (T1) and commercial *S. cerevisiae* and water kefir (T2), respectively, while values of 6.69 and 10.84% were observed in this study, respectively. These small variations may be related to factors such as the amount of must used in 5 L fermentation buckets, since Souza et al. [6] produced 2.5 L, while in this study 3 L were produced. In addition, it is noteworthy that Souza et al. [6] needed to take samples of the product in order to monitor the fermentation kinetics, whereas in the present study, fermentation occurred continuously. However, it should be noted that the results observed for both studies are similar.

**Figure 2:**
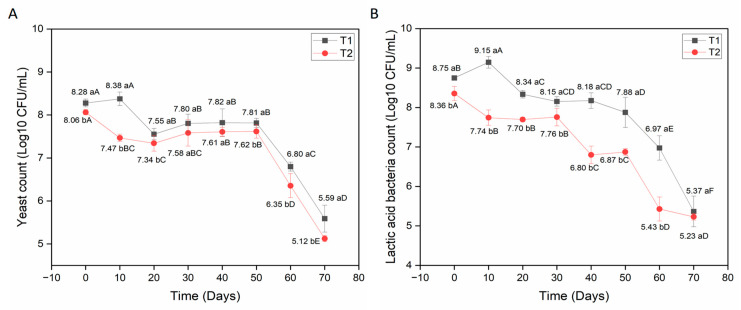
Viable cell count in mead during storage at 7 °C ± 1 °C. (**A**) Yeast count (Log CFU/mL) and (**B**) Lactic acid bacteria count (Log CFU/mL). T1 = mead with kefir and *S. boulardii*; T2 = mead with kefir and commercial *S. cerevisiae*. Different lowercase letters indicate significant differences between treatments on the same day. Different uppercase letters indicate significant differences for each specific treatment and throughout the storage period (according to Tukey’s test, *p* < 0.05). All analyses were performed in triplicate.

With regard to contaminating microorganisms, no total coliform counts, *E. coli* counts, or *Salmonella* spp. counts were observed in the mead during refrigerated storage. It should be noted that no contaminants were expected to be found in the finished product, since the must was initially pasteurized in order to eliminate possible pathogens. However, these analyses were important to show that during the product’s shelf life there was no interference from undesirable microorganisms, as well as to testify that all processing and production of the mead took place under adequate sanitary conditions, preventing even possible cross-contamination. Thus, the results were negative for all contaminating microorganisms analyzed. The results for the viable yeast and lactic acid bacteria counts in the mead are shown in Figure 2.

According to Figure 2A, mead with *S. boulardii* and water kefir (T1) has a higher yeast count, with a significant difference (*p* < 0.05) compared to mead with commercial *S. cerevisiae* and water kefir (T2) at the beginning and end of storage. It is also observed that the yeast count in T1 decreased by approximately 2.69 Log CFU/mL and in T2 decreased by 2.94 Log CFU/mL, with a significant difference (*p* < 0.05) when comparing the initial (0 days) and final (70 days) storage times of the product. On the other hand, the LAB count (Figure 2B) increased by 0.4 Log CFU/mL in T1, when observing the initial time (0 days) and 10 days of storage, followed by a reduction of 3.78 Log CFU/mL up to 70 days of storage. With regard to T2, a reduction in LAB of approximately 3.13 Log CFU/mL was observed when comparing the initial time (0 days) and the end of 70 days of storage. The reduction in yeast viability and LAB in mead during refrigerated storage was expected due to the increase in alcohol content. Even in smaller quantities, microorganisms are exposed to alcoholic stress, favoring cell death. Studies in the literature corroborate that ethanol in high concentrations can reduce vitality and increase cell death [6,10,26,27], with fermented alcoholic beverages constituting a high-stress environment for the maintenance of probiotic microorganisms [15].

It can be observed in Figure 2A,B that up to 60 days of storage, mead with *S. boulardii* and water kefir (T1) still had microorganism counts above 6 Log CFU/mL, while at 70 days, the counts dropped to 5 Log CFU/mL. Therefore, considering that 6 Log CFU/mL is considered the minimum therapeutic dose for probiotic products [6,10,27,28,29,30], it can be inferred that 60 days of storage at 7 ± 1 °C represents the most appropriate shelf life for potentially probiotic mead obtained by mixed fermentation of *S. boulardii* and water kefir (T1).

**Figure 3:**
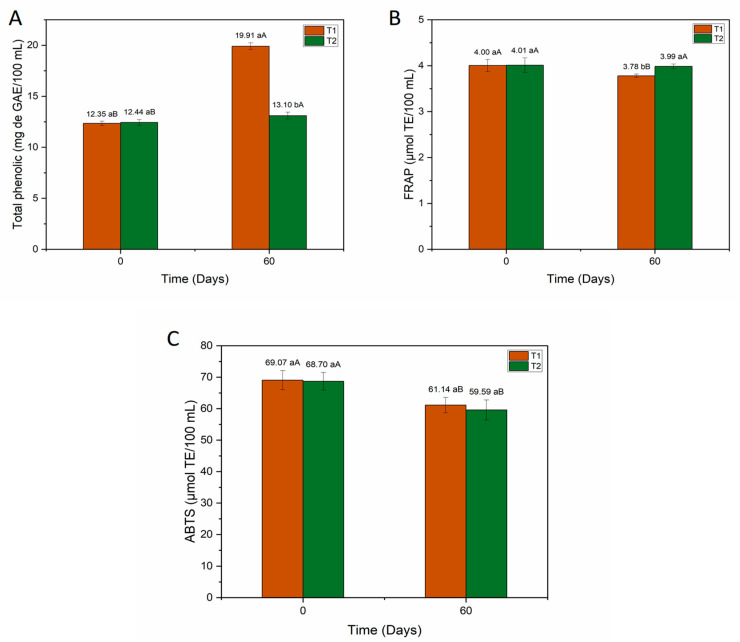
Bioactive compounds in mead during storage at 7 °C ± 1 °C. (**A**) Total phenolics (mg GAE/100 mL), (**B**) Antioxidants by FRAP (µmol TE/100 mL), and (**C**) Antioxidants by ABTS (µmol TE/100 mL). T1 = mead with kefir and *S. boulardii*; T2 = mead with kefir and commercial *S. cerevisiae*. Different lowercase letters indicate significant differences between treatments on the same day. Different uppercase letters indicate significant differences for each specific treatment and throughout the storage period (according to Tukey’s test, *p* < 0.05). All analyses were performed in triplicate.

The analysis of bioactive compounds in mead was performed considering a shelf life of 60 days of refrigerated storage, since during this storage period the product still had a minimum microorganism count of 6 Log CFU/mL for probiotic products. The results of the bioactive compounds are shown in Figure 3. According to Figure 3A, mead with *S. boulardii* and water kefir (T1) and commercial *S. cerevisiae* and water kefir (T2) have statistically equal amounts of phenolic compounds (*p* < 0.05) at the initial time. However, after 60 days of storage, there is a significant increase (*p* < 0.05) in phenolic compounds of 7.56 mg GAE/100 mL in T1 and 0.66 mg GAE/100 mL in T2. For antioxidants, there is a significant reduction (*p* < 0.05) of 0.22 μmol TE/100 mL of antioxidants by FRAP in T1, although there was no change in the antioxidant content by FRAP in T2 (Figure 3B). With regard to antioxidants by ABTS (Figure 3C), a significant reduction (*p* < 0.05) of 7.93 μmol TE/100 mL was observed in T1 and 9.11 μmol TE/100 mL in T2. The results for bioactive compounds in mead reveal a possible synergy between *S. boulardii* and water kefir favoring the production of phenolic compounds during storage, although there is a loss and decrease in antioxidant capacity during refrigerated storage for 60 days. As shown in Figure 3A, there was an abrupt increase in total phenolics for T1 during storage and a decline in antioxidant activity for the FRAP (Figure 3B) and ABTS (Figure 3C) methods, which was unexpected. However, it has been reported in the literature that an increase in the content of phenolic compounds concomitant with a reduction in antioxidant activity in alcoholic beverages can occur during the aging process [31]. At this stage, chemical transformations—especially spontaneous oxidative reactions—can modify the structure of phenolics, resulting in partial or total loss of their reducing capacity. Thus, even though the total concentration of phenolics increases, antioxidant activity tends to decrease because oxidation alters the molecular conformation of these compounds, compromising their antioxidant functionality. According to Adamenko et al. [31], who studied the production of mead for 14 days at a temperature of 22 °C and maturation for 3 months at a temperature of 8 °C, the antioxidant activity of meads may decrease due to alcoholic fermentation and also the aging process. However, future studies should be conducted to gain a clearer understanding of the increase in phenolic compounds during the storage of mead with *S. boulardii* and water kefir, as well as the identification and characterization of such components.

We can highlight in this study that, in general, the results for phenolics and antioxidants corroborate studies in the literature that have demonstrated mead to be a fermented alcoholic beverage that is a source of bioactive compounds [1,3,4,6,31]. Thus, it should be noted that the presence of antioxidants in mead is important because of the ability of these compounds to inhibit free radicals that can cause cell damage [6,14,32].

### 3.2. Sensory Analysis Results

#### 3.2.1. Screening of Tasters

**Figure 4:**
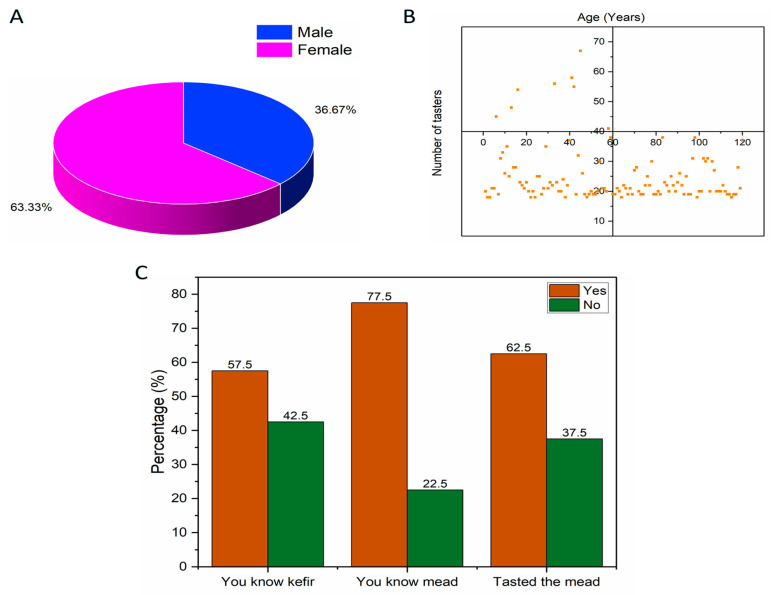
Profile and general characteristics of sensory analysis tasters. (**A**) Gender of tasters, (**B**) Age group of tasters, and (**C**) Level of knowledge about the products.

A total of 120 tasters participated in the sensory evaluation. Figure 4 shows a screening of the profile and general characteristics of the sensory analysis tasters. Regarding gender, approximately 36.67% (total of 44 tasters) were male and 63.33% (total of 76 tasters) were female, according to Figure 4A. The general age range of the participants varied from 18 to 67 years, with most tasters falling within the 18 to 28 age range (Figure 4B). For an initial assessment, when asked if they were familiar with kefir and mead prior to sensory analysis, approximately 57.5% (total of 69 tasters) were familiar with kefir, while 77.5% (total of 93 tasters) said they were familiar with mead, as shown in Figure 4C. When asked if they had tasted mead before the sensory analysis, about 62.5% (total of 75 tasters) said they had tried the product on some occasion. This initial assessment showed that most of the sensory analysis tasters were young, most of whom were already familiar with kefir and mead and had tasted the product on some occasion.

#### 3.2.2. Product Acceptance and Purchase Intention

Table 1 shows the results of sensory acceptance and purchase intention for mead. According to Table 1, mead with *S. boulardii* and water kefir (T1) showed greater acceptance for the attributes of overall impression, flavor, and alcohol content, with a significant difference (*p* < 0.05) compared to mead with commercial *S. cerevisiae* and water kefir (T2) and mead sold on the domestic market (T3). For the attributes color and aroma, T1 was statistically equal to T2 and different from T3. These results show that mead by *S. boulardii* and water kefir (T1) was more accepted, corroborating other studies in the literature that showed good acceptance of alcoholic beverages fermented by probiotic yeast *S. boulardii* [12,25,33,34].

With regard to purchase intention (Table 1), mead with *S. boulardii* and water kefir (T1) had the highest score, with a significant difference (*p* < 0.05) compared to commercial mead with *S. cerevisiae* and water kefir (T2) and mead sold on the domestic market (T3), although T2 and T3 are statistically equal. These results for purchase intention corroborate the acceptance results, since T1 was more accepted and, consequently, showed a higher purchase intention by the evaluators. Souza et al. [12], studying the sensory characterization of mead with different soluble solids in the initial must, also demonstrated good acceptance and purchase intention for products made by *S. boulardii*. It is also worth noting that favorable purchase intention for a product can generate a higher probability of purchase, while an unfavorable attitude can generate a lower probability of use [12,35]. Thus, these results demonstrate a greater possibility of use and purchase of mead by *S. boulardii* and water kefir, as demonstrated in the acceptance and purchase intention of the products.

#### 3.2.3. Check-All-That-Apply (CATA) Test Results

**Figure 5:**
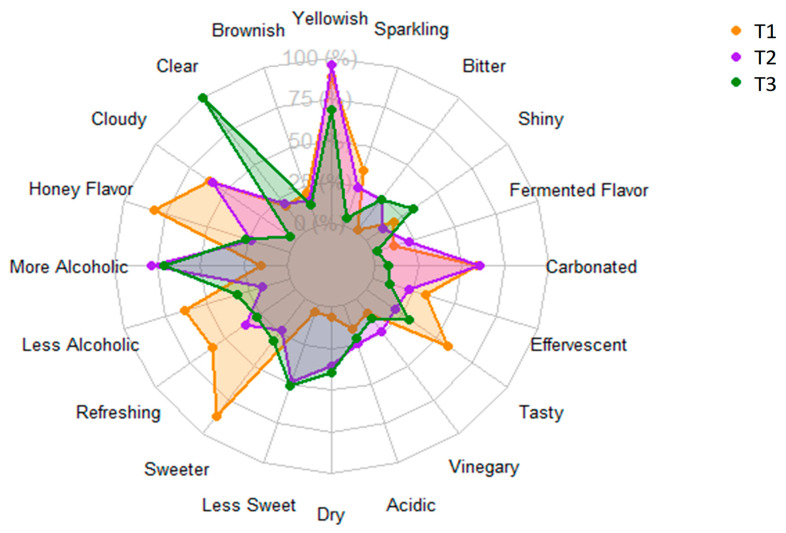
Frequency of selection of descriptive terms in the CATA questionnaire for meads. T1 = mead with kefir and *S. boulardii*; T2 = mead with kefir and commercial *S. cerevisiae*; T3 = mead sold on the domestic market, a drink produced with eucalyptus flower honey. The differences between treatments were significant according to the Chi-square test for homogeneity (*p* < 0.05).

With regard to the frequency of descriptive terms in the CATA questionnaire, the results are shown in Figure 5. According to CATA, mead with *S. boulardii* and water kefir (T1) was mainly associated with the terms effervescent, tasty, sweeter, refreshing, honey flavor, and less alcoholic, which is corroborated by the lower alcohol content observed for T1, shown in Figure 1D. On the other hand, T2 presented characteristics described as more alcoholic, yellowish, fermented flavor, and carbonated, while T3 presented characteristics described for a shiny, dry, less sweet, and clear product. In this sense, it can be inferred that these sensory characteristics frequently described by the panelists contributed to characterizing greater acceptance and purchase intention of mead by *S. boulardii* and water kefir (T1), compared to the other products.

We can also highlight that although commercial mead on the domestic market (T3) has an alcohol content of 13% on its label, tasters noted T2 as a slightly more alcoholic product, possibly related to the complexity and interaction of the sensory attributes described for T2, translating into a perception of higher alcohol content. In addition, T3 was strongly characterized as clear, demonstrating that despite the increasing costs of production and industrial processing stages, some production steps, such as clarification, filtration, and centrifugation to remove yeast cells, are included in the process and considered important for industrial products intended for sale in regional, domestic, and international markets [7,36].

In this study, some aspects should be considered for a more consistent interpretation of the results. Although we did not evaluate the survival of microorganisms in simulated gastrointestinal conditions after storage, it was observed that the product maintained, at the end of 60 days, the minimum recommended count of 6 Log CFU/mL for probiotic products. Despite the absence of specific regulations for alcoholic beverages containing probiotics, it should be noted that alcohol consumption should follow international recommendations, such as those of the World Health Organization (WHO), which establishes 10 g of pure ethanol as the standard daily dose, equivalent to 250 mL of beer, 230 mL of cider, 100 mL of wine, or 31 mL of spirits [37]. Furthermore, the literature indicates that daily intake between 8 and 9 Log CFU/mL is necessary to obtain physiological benefits [27,38]. Thus, our results contribute to the advancement of knowledge related to the development, labeling, and market positioning of potentially probiotic alcoholic beverages, signaling their possible insertion into functional product niches.

Some limitations must be considered, including the need to evaluate the survival of microorganisms in simulated gastrointestinal conditions and to characterize volatile and non-volatile compounds throughout the shelf life for better sensory understanding. In addition, alcoholic beverages with probiotics face technological challenges, such as low ethanol tolerance and difficulty in maintaining viable counts, as well as marketing and regulatory barriers due to the ambiguous nature of the product between alcoholic and functional. Although they may present environmental opportunities, additional processing steps increase complexity and environmental footprint. From a public health perspective, there is a contradiction between the potential benefits of probiotics and the risks inherent in alcohol consumption. Thus, despite its innovative potential, this type of product requires technological advances and carefully considered market positioning.

## 4. Conclusions

The results of this study demonstrate that storing mead at 7 °C ± 1 °C does not completely inhibit residual fermentation activity, showing that microorganisms present in the system remain metabolically active and continue to produce ethanol, albeit at reduced levels. The shelf life assessment confirmed that mead obtained by mixed fermentation with *Saccharomyces boulardii* and water kefir maintains viable yeast and lactic acid bacteria counts above 6 Log CFU/mL for 60 days, meeting the minimum limit established for products with potential probiotic claims. In terms of sensory characteristics, the formulation was highly accepted and showed high purchase intent, being associated with positive attributes such as effervescence, balanced sweetness, refreshment, honey notes, and lower alcohol perception, indicating that the fermentation combination used results in a distinctive sensory profile that is attractive to consumers.

Together, these findings confirm the potential of mead made with *S. boulardii* and water kefir as an innovative alternative in the fermented alcoholic beverage segment, combining technological, functional, and sensory properties. Therefore, although the product has strong technological and commercial potential, its development and future industrial application require process optimization, in-depth functional and aromatic analyses, and the definition of scientifically based marketing and regulatory strategies to ensure safety, efficacy, and adequate positioning in the functional beverage market.

## Figures and Tables

**Table 1:** Acceptance and purchase intention of mead.

Attributes	T1	T2	T3
Overall impression	7.78 ± 1.15 a	6.85 ± 1.59 b	6.27 ± 2.13 c
Color	7.34 ± 1.41 a	7.11 ± 1.42 a	6.53 ± 2.11 b
Aroma	7.13 ± 1.58 a	6.92 ± 1.67 ab	6.53 ± 1.83 b
Flavor	7.95 ± 1.23 a	6.40 ± 1.92 b	6.00 ± 2.31 b
Alcohol content	7.47 ± 1.51 a	6.78 ± 1.75 b	6.27 ± 1.92 c
Purchase intention	5.17 ± 1.39 a	4.07 ± 1.62 b	3.68 ± 1.86 b

T1 = mead with kefir and *S. boulardii*; T2 = mead with kefir and commercial *S. cerevisiae*; T3 = mead sold on the domestic market, a drink produced with eucalyptus flower honey. Lowercase letters on the same line indicate a significant difference between treatments, according to Tukey’s test (*p* < 0.05).

## Data Availability

The original contributions presented in this study are included in the article. Further inquiries can be directed to the corresponding author.
